# OpenSFDI: an open-source guide for constructing a spatial frequency domain imaging system

**DOI:** 10.1117/1.JBO.25.1.016002

**Published:** 2020-01-10

**Authors:** Matthew B. Applegate, Kavon Karrobi, Joseph P. Angelo Jr., Wyatt Austin, Syeda M. Tabassum, Enagnon Aguénounon, Karissa Tilbury, Rolf B. Saager, Sylvain Gioux, Darren Roblyer

**Affiliations:** a Boston University, Department of Biomedical Engineering, Boston, Massachusetts, United States; b University of Strasbourg, ICube Laboratory, Strasbourg, France; c University of Maine, Department of Chemical and Biomedical Engineering, Orono, Maine, United States; d Boston University, Department of Electrical and Computer Engineering, Boston, Massachusetts, United States; e Linköping University, Department of Biomedical Engineering, Linköping Sweden

**Keywords:** spatial frequency domain imaging, modulated imaging, diffuse optics, frequency domain, optical properties, open source

## Abstract

**Significance**: Spatial frequency domain imaging (SFDI) is a diffuse optical measurement technique that can quantify tissue optical absorption ($\mu_{a}$) and reduced scattering ($\mu_{s}^{\prime }$) on a pixel-by-pixel basis. Measurements of $\mu_{a}$ at different wavelengths enable the extraction of molar concentrations of tissue chromophores over a wide field, providing a noncontact and label-free means to assess tissue viability, oxygenation, microarchitecture, and molecular content. We present here openSFDI: an open-source guide for building a low-cost, small-footprint, three-wavelength SFDI system capable of quantifying $\mu_{a}$ and $\mu_{s}^{\prime }$ as well as oxyhemoglobin and deoxyhemoglobin concentrations in biological tissue. The companion website provides a complete parts list along with detailed instructions for assembling the openSFDI system.

**Aim**: We describe the design of openSFDI and report on the accuracy and precision of optical property extractions for three different systems fabricated according to the instructions on the openSFDI website.

**Approach**: Accuracy was assessed by measuring nine tissue-simulating optical phantoms with a physiologically relevant range of $\mu_{a}$ and $\mu_{s}^{\prime }$ with the openSFDI systems and a commercial SFDI device. Precision was assessed by repeatedly measuring the same phantom over 1 h.

**Results**: The openSFDI systems had an error of 0±6% in $\mu_{a}$ and −2±3% in $\mu_{s}^{\prime }$, compared to a commercial SFDI system. Bland–Altman analysis revealed the limits of agreement between the two systems to be $\pm 0.004\;{\mathrm{mm}}^{-1}$ for $\mu_{a}$ and −0.06 to $0.1\;{\mathrm{mm}}^{-1}$ for $\mu_{s}^{\prime }$. The openSFDI system had low drift with an average standard deviation of $0.0007\;{\mathrm{mm}}^{-1}$ and $0.05\;{\mathrm{mm}}^{-1}$ in $\mu_{a}$ and $\mu_{s}^{\prime }$, respectively.

**Conclusion**: The openSFDI provides a customizable hardware platform for research groups seeking to utilize SFDI for quantitative diffuse optical imaging.

## 1 Introduction

Spatial frequency domain imaging (SFDI) is a noninvasive, label-free, diffuse optical technique that can generate two-dimensional maps of the absorption coefficient ($\mu_{a}$) and reduced scattering coefficient ($\mu_{s}^{\prime }$) of biological tissue. To accomplish this, SFDI utilizes sinusoidally modulated illumination patterns, which are projected onto the sample, and the remitted light is detected with a camera. A demodulation and calibration procedure is used to estimate the spatial modulation transfer function (MTF) of the sample. The MTF can then be fit to a computational model of photon transport to determine both $\mu_{a}$ and $\mu_{s}^{\prime }$ on a pixel-by-pixel basis.^1^ Knowledge of $\mu_{a}$ at several different carefully chosen wavelengths allows for the molar concentration of tissue chromophores such as oxyhemoglobin and deoxyhemoglobin to be estimated. Both optical properties and chromophore concentrations can then be used as optical biomarkers for a wide range of applications.

The ability of SFDI to provide quantitative physiological and molecular information in tissue, combined with its relative simplicity, safety, and low cost, has led to its use for a variety of biomedical applications. These include perfusion monitoring of skin flaps during graft surgery,^2^^–^^4^ investigations of blood flow in the brain,^5^ and assessment of resected tissues following surgery.^6^^,^^7^ The relatively shallow penetration depth of SFDI (typically less than 5 mm)^8^ makes this technique particularly well suited for assessing skin conditions such as port wine stain birthmarks,^9^^,^^10^ burns,^11^^–^^13^ scarring,^14^ pressure ulcers,^15^ nonmelanoma skin cancer,^16^ and diabetic vascular disease.^17^ SFDI has been used in the preclinical setting to noninvasively monitor tumor hemodynamics during treatment *in vivo*.^18^ SFDI has also been used in nonbiomedical applications, including the assessment of fruit quality in apples^19^ and peaches.^20^ For more details regarding the theory and applications of SFDI, we direct readers to two excellent recent reviews.^21^^,^^22^

**Fig. 1:**
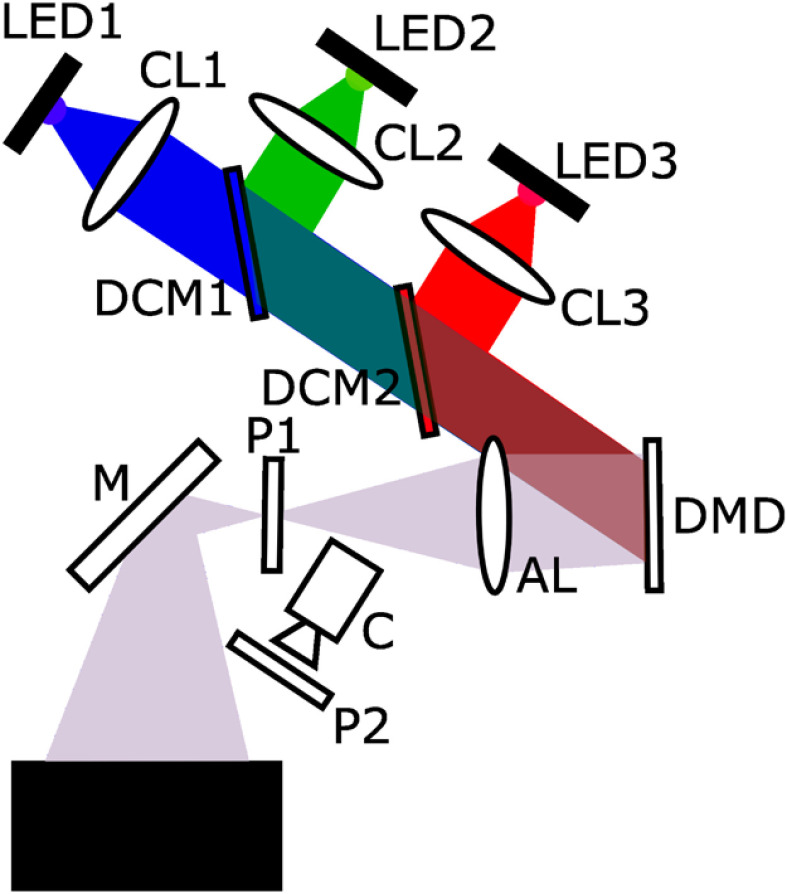
Schematic representation of the openSFDI system. CL, collimating lens; DCM, dichroic mirror; AL, achromatic lens; P, linear polarizers; M, mirror; C, camera; DMD, digital micromirror device.

The goal of this paper and associated website is to provide a complete guide for constructing an SFDI device (shown schematically in Fig. 1), acquiring SFDI data, and determining optical properties in tissue. In the following sections, we describe the key components of the openSFDI system, outline the data-processing pipeline, and present results of the characterization of three openSFDI systems that have recently been constructed. Our hope is that the openSFDI project will reduce the barriers for research groups interested in incorporating SFDI into their research programs.

## 2 Spatial Frequency Domain Imaging Hardware

**Fig. 2:**
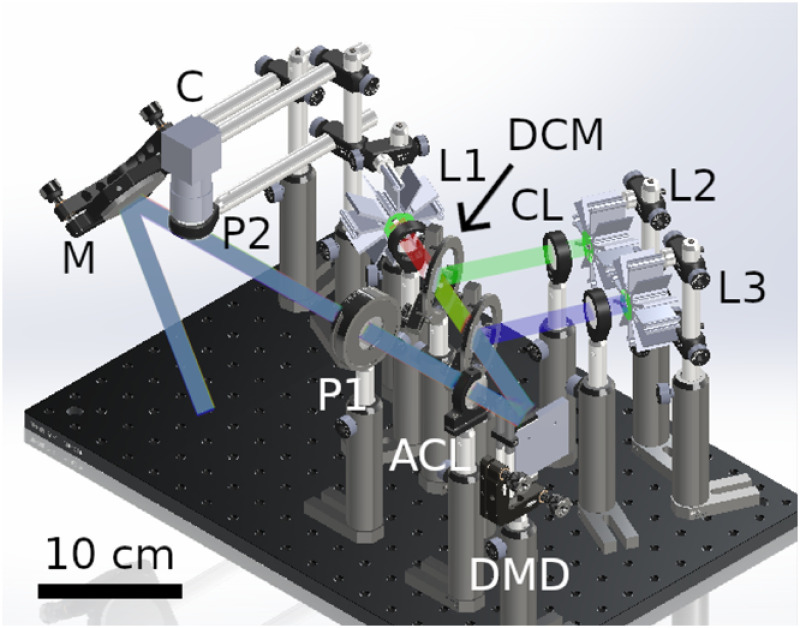
CAD rendering of the openSFDI system with the light paths for the three wavelengths. L, light-emitting diode; CL, collimating lens (only one of three labeled); DCM, dichroic mirror (only one of two indicated); ACL, achromatic lens; P, linear polarizers; M, mirror; C, camera; DMD, digital micromirror device.

There are three main components to an SFDI system: the light source, the method of spatially modulating the illumination field, and the detector. The specific components of the openSFDI system were chosen to balance availability, cost, and overall performance. A rendering of the openSFDI system that includes all the components is shown in Fig. 2. Each openSFDI component will be discussed in the following subsections, followed by an analysis of the overall system performance. A complete materials list is available at Ref. 23. Whenever possible, components were sourced from commonly used suppliers in the United States to make the purchasing process easier. At the time of this writing, the total cost of an openSFDI system was US$4717.

### 2.1 Illumination Source

The first published SFDI systems used a broadband mercury lamp as a light source combined with a tunable emission filter for wavelength selection.^1^ Subsequent systems have commonly utilized laser diodes or an array of light-emitting diodes (LEDs) for illumination, eliminating the need for filtering.^24^^,^^25^ OpenSFDI uses three high-powered near-infrared LEDs with center wavelengths of 660, 735, and 865 nm. These wavelengths were chosen to straddle the isosbestic point of oxyhemoglobin and deoxyhemoglobin at 800 nm to enable high sensitivity to changes in these chromophores. We note that LED center wavelengths can vary significantly, even for LEDs fabricated from a single manufacturer. Accurate knowledge of the illumination wavelength is critical to ensure an accurate extraction of chromophores from the sample. To account for variability in LED wavelength, we recommend that a spectrometer be used to characterize the center wavelength of each LED used in the openSFDI system and that subsequent data-processing steps assume the measured rather than manufacturer specified wavelength. In addition, LED optical output power can be unstable due to thermal effects and/or instability of the driving current. OpenSFDI utilizes heatsinks to stabilize the temperature of the LEDs, but we recommend that the stability of each LED source be tested and characterized for each system (see Sec. 5.2). This step is particularly important if the target application includes longitudinal repeated measurements or long exposure times. Both of these scenarios can lead to LED temperature fluctuations resulting in unstable light output.

### 2.2 Spatial Modulation

Spatially modulated illumination is required to measure the MTF of the sample for SFDI. A simple method to generate illumination patterns is to pass light through a suitably patterned optical transparency.^26^^,^^27^ However, this method lacks the flexibility to rapidly alter the illumination pattern, reducing its utility for many applications. Most published SFDI systems utilize digital micromirror devices (DMDs) to project patterns onto the sample. DMDs have an array of mirrors that can be individually addressed and tilted to direct and pattern the illumination field. The DMD chosen for openSFDI (LC4500, Keynote Photonics) is available as a small DMD chipset with a 0.45" WXGA resolution DMD. The DMD controller is provided on a separate printed circuit board. The software interface provides low-level access to the DMD hardware which is critical to ensure that no automatic corrections are applied to the input image. For example, gamma correction, which is commonly used to improve image quality for human visualization, can result in unwanted harmonics in the projected sinusoidal pattern and lead to demodulation issues. The openSFDI website provides additional examples of distorted projections as well as instructions for troubleshooting these issues.^28^

### 2.3 Detection

The camera selected for openSFDI (BlackFly-S BFS-U3-13Y3M-C, FLIR, Wilsonville, Oregon) is a 1280×1024  pixel, 10-bit, monochromatic CMOS camera chosen because it is relatively low cost and can easily interface with LabVIEW. In addition, it allows for low-level control of the image acquisition which makes it possible to turn off automatic corrections that might lead to nonlinear output. We have also constructed openSFDI systems using scientific CMOS (sCMOS) cameras, as discussed in later sections. Crossed linear polarizers are used in the system to limit the effect of specular reflection from the sample.

## 3 Acquisition

OpenSFDI was designed to make it easy to rapidly begin acquiring SFDI data. To that end, the website provides LabVIEW acquisition code that will run out of the box with any camera supported by IMAQdx. With modifications, the software may also function with cameras supported by the older IMAQ infrastructure. The software has options for collecting multiple measurements at user-defined intervals, selecting wavelengths, choosing spatial frequencies, and allows users to choose the orientation of the spatially projected patterns. The pattern orientation is critical for making profilometric measurements (see Sec. 5.3.2).

The openSFDI system will produce an illuminated area of about 7.5×4.5  cm, if the instructions on the website are followed closely. The working distance between the sample plane and the detector as well as the field of view depends on the choice of lens mounted to the camera. At an aperture of f/4, typical exposure times are on the order of 100 ms per image depending on the sample and wavelength.

## 4 Data Processing

### 4.1 Calibration

Most diffuse optical techniques, including SFDI, rely on a calibration measurement to determine the instrument response function (IRF). Once known, the IRF can be removed from subsequent measurements, a step needed to obtain accurate estimates of $\mu_{a}$ and $\mu_{s}^{\prime }$. Calibration is typically performed using a tissue-mimicking optical phantom with well characterized $\mu_{a}$ and $\mu_{s}^{\prime }$ at the illumination wavelengths. Data taken on the calibration phantom are compared with the theoretical diffuse reflectance ($R_{d}$) values associated with the known calibration phantom optical properties to calculate the IRF. A forward model of photon propagation that maps $\mu_{a}$ and $\mu_{s}^{\prime }$ to $R_{d}$ in the spatial frequency domain is used to obtain the theoretical calibration $R_{d}$ values and determine the IRF.

Accurate characterization of the calibration phantom is critical to obtaining accurate SFDI results. Optical phantoms can be fabricated by adding an absorbing agent and a scatterer to an optically neutral substrate such as silicone^29^^,^^30^ Time-resolved and frequency-domain diffuse optical measurements, as well as inverse adding doubling, are generally considered accurate methods for determining the optical properties of a calibration phantom.^31^^,^^32^ Some works have utilized intralipid as a calibration standard, as it is subject to a highly controlled manufacturing environment, and both $\mu_{a}$ and $\mu_{s}^{\prime }$ of standard intralipid emulsions have been characterized in literature.^33^^,^^34^ We note that characterized calibration phantoms may be purchased from the Institut National d’Optique (Biomimic^™^),^35^ but we have not tested or utilized these phantoms for this work. New calibration phantoms may also be characterized with existing SFDI devices, provided the system was adequately calibrated using a known standard. Calibration phantoms produced in this way may suffer from the propagation of measurement errors. The calibration phantom used in this study was provided and characterized by Modulim Inc. (Irvine, California). It had dimensions of 25×25×3  cm (L×W×H) and a $\mu_{a}$ of $0.007\;{\mathrm{mm}}^{-1}$ and a $\mu_{s}^{\prime }$ of $0.93\;{\mathrm{mm}}^{-1}$ at 800 nm.

### 4.2 Optical Property Estimates

The procedure for estimating optical properties from SFDI data has been previously described in detail.^1^ Briefly, sinusoidal illumination patterns are projected onto the sample at three phases (0, π/3, and 2π/3). The resulting collected images are then computationally demodulated to isolate the tissue response function at the projected spatial frequency. The IRF is removed by comparison with a calibration phantom (Sec. 4.1) to calculate the $R_{d}$ as a function of spatial frequency (i.e., the MTF). Measurement of the MTF at two or more spatial frequencies allows the $\mu_{a}$ and $\mu_{s}^{\prime }$ of the sample to be calculated on a pixel-by-pixel basis. The choice of spatial frequencies for a given application is multifaceted and depends on the optical properties of the sample^36^ and the partial volume effects caused by using both a low spatial frequency (which is generally more deeply penetrating) and a high spatial frequency (which is generally less deeply penetrating) together for a single SFDI measurement.^8^ Our lab uses DC and $0.1\;{\mathrm{mm}}^{-1}$ for several of our projects,^18^ though the use of AC frequencies up to about $0.2\;{\mathrm{mm}}^{-1}$ are also common.^37^^,^^38^ The openSFDI website provides a step-by-step guide to developing an SFDI processing pipeline,^39^ as well as an executable application for validating processing code written in-house. In addition, the code utilized to generate the figures in this paper is available upon request.

**Fig. 3:**
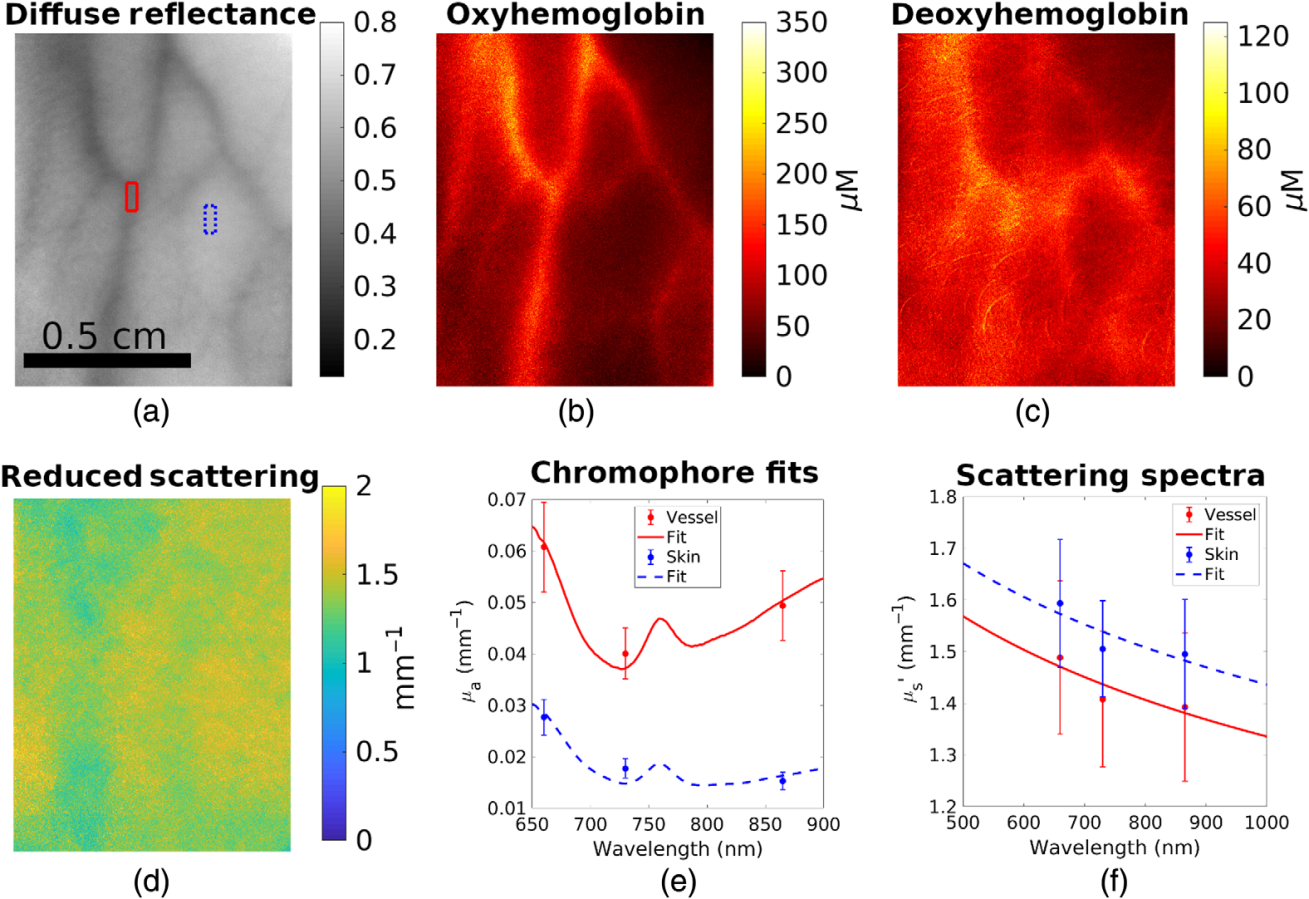
OpenSFDI results in biological tissue. (a) Diffuse reflectance of the back of a hand at 865 nm. Superficial vasculature is apparent due to hemoglobin absorption. Boxes indicate regions of interest for the line plots. (b) Estimated concentration of oxyhemoglobin. (c) Estimated concentration of deoxyhemoglobin. (e) Image of $\mu_{s}^{\prime }$ at 865 nm. (f) Absorption spectra (points) and chromophore fits for the vascular compartment (red solid line) and nonvascular compartment (blue dashed line). (g) Scattering spectra (points) and power law fits (lines) for the vascular and nonvascular compartments. Error bars represent ±1 standard deviation of the pixels in the regions of interest. Scale bar applies to all images.

Knowledge of $\mu_{a}$ at several wavelengths allows the concentration of tissue chromophores to be calculated. Using extinction spectra of oxyhemoglobin and deoxyhemoglobin from literature,^40^ the concentration of each of these chromophores can be determined by finding the linear combination of concentrations that most closely match the collected data. This calculation is done by solving a system of linear equations. An example of openSFDI images of the back of a hand *in vivo* is shown in Fig. 3.

## 5 Characterization

### 5.1 Accuracy

The accuracy of three different openSFDI systems was assessed by comparing them with a commercial SFDI device (Reflect RS, Modulim, Irvine, California). Nine tissue-mimicking optical phantoms containing different amounts of nigrosin and titanium dioxide nanoparticles, to tune absorption and scattering, respectively,^41^ were measured by each system at spatial frequencies of 0 and $0.1\;{\mathrm{mm}}^{-1}$. The phantoms were fabricated to have absorption and scattering that spanned a physiologically relevant range ($0.005<\mu_{a}<0.07\;{\mathrm{mm}}^{-1}$ and $0.5<\mu_{s}^{\prime }<2\;{\mathrm{mm}}^{-1}$). Measurements were repeated three times for each instrument, and all the raw data were processed identically. Optical properties were estimated from diffuse reflectance values using a two spatial frequency lookup table generated from Monte Carlo simulations.^42^

The three systems followed the same overall design as the schematic in Fig. 1 but used different cameras. The first system used a FLIR BlackFly-S camera (FLIR, Wilsonville, Oregon), the second system utilized an Andor sCMOS camera (Zyla, Oxford Instruments, Oxford), and the third system used a Thorlabs sCMOS device (Quantalux, Thorlabs, Newton, New Jersey). The first system was constructed at Boston University by S.M.T. and M.B.A., the second system was also fabricated at Boston University by K.K., while the third was initially constructed at the University of Maine by W.A. and K.T. and transported to Boston University for characterization.

Interpolation of $\mu_{a}$ and $\mu_{s}^{\prime }$ values from the commercial instrument was conducted to account for the fact that the wavelengths used for openSFDI (660, 735, and 865 nm) were different from the commercial device (621, 691, 731, 811, and 851 nm). A cubic spline was used to fit the absorption spectrum measured by the commercial system and $\mu_{a}$ values at the openSFDI wavelengths were found via interpolation. For $\mu_{s}^{\prime }$, the scattering spectrum measured by the commercial device was fit to the equation $\mu_{s}^{\prime }={a(\lambda /\lambda_{0})}^{-b}$, where λ represents the wavelength, $\lambda_{0}$ was set at 800 nm, and a and b were constants. Different a and b values were found for each phantom. The $\mu_{s}^{\prime }$ values for the openSFDI wavelengths were found by substituting λ into the fit equation for each wavelength on each phantom.

**Fig. 4:**
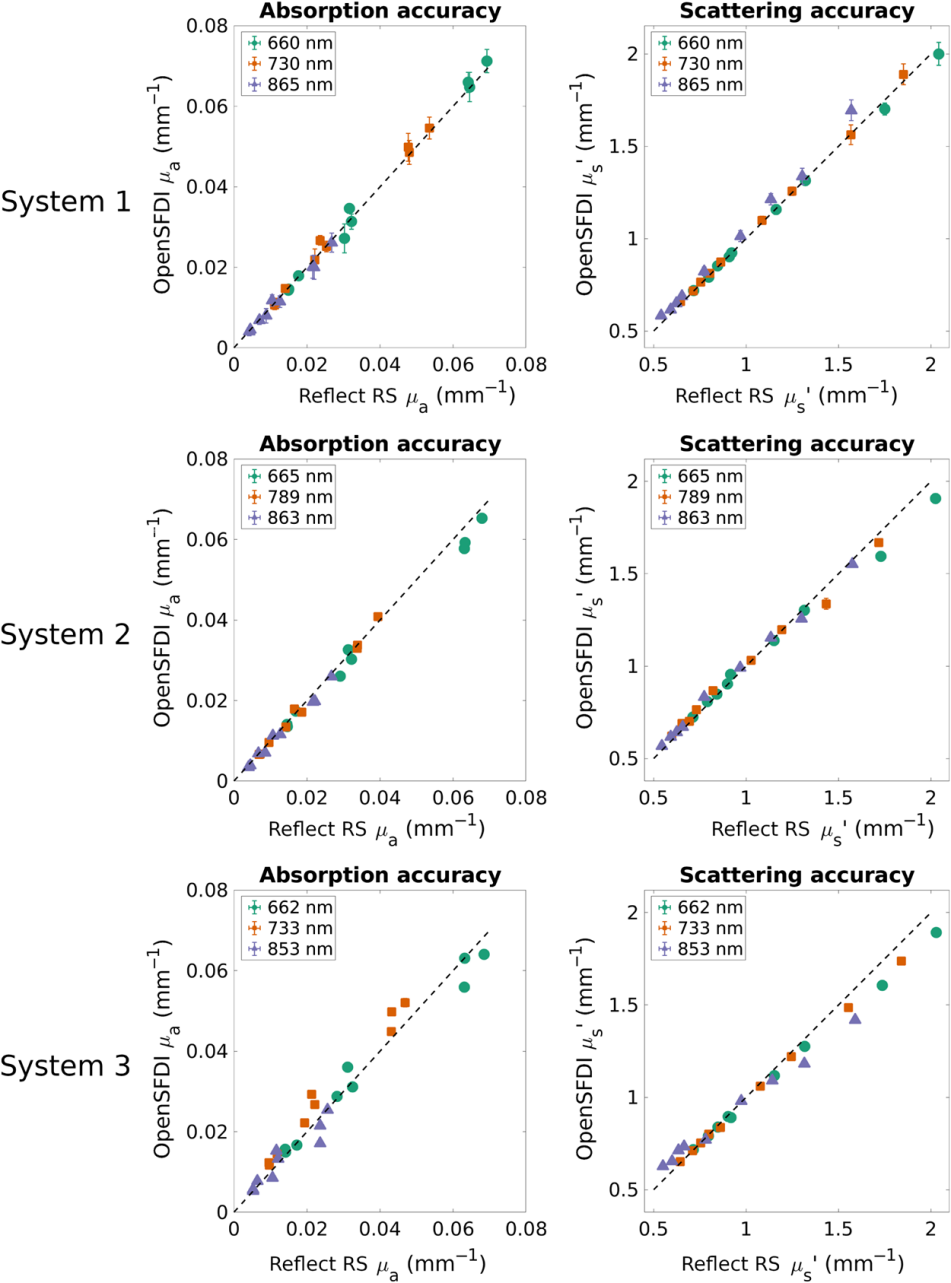
Accuracy of three openSFDI systems compared with a commercial SFDI device. Data points represent the average optical property of a phantom measured three times. Error bars are the standard deviation across the three measurements. System 1 was constructed at Boston University and used an FLIR CMOS camera. System 2 was constructed at Boston University and used an Andor sCMOS camera. System 3 was constructed at the University of Maine and used a Thorlabs sCMOS camera. The average ± standard deviation difference between openSFDI and the commercial system was 0±6%, 4±6%, and −7±15% for $\mu_{a}$ and −2±3%, −1±4%, and 1±6% for $\mu_{s}^{\prime }$, respectively. Note that each system uses slightly different wavelengths due to variations in LED manufacturing. Error bars on the bottom plots are similar in size to the data points.

Comparisons between the systems were made by averaging the pixels within a 1×2  cm region of interest from each optical property map. The average optical properties for each of the three measurements is plotted in Fig. 4. The difference in $\mu_{a}$ between the openSFDI systems and the commercial system was 0±6%, 4±6%, and −7±15% for the FLIR-, Andor-, and Thorlabs-based systems respectively, while the difference in $\mu_{s}^{\prime }$ was found to be −2±3%, −1±4%, and 1±6%, respectively. As evidenced by the larger vertical error bars in the top row of Fig 4, the FLIR system had more variation than the Andor or Thorlabs systems. This variability is likely due to the fact that the FLIR sensor is not cooled.

**Fig. 5:**
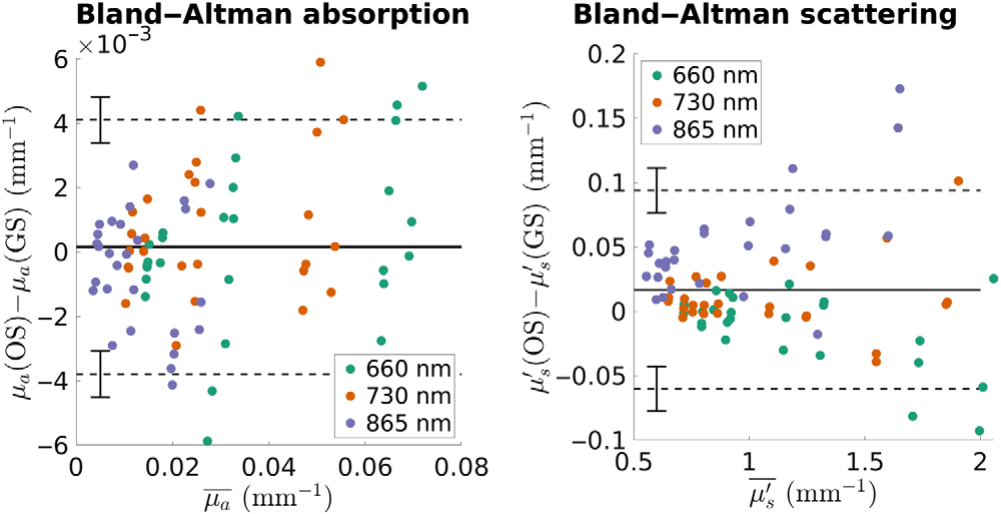
Bland–Altman plots comparing openSFDI (OS) with a commercial, gold standard (GS), SFDI system. The horizontal axis is the average of the two instruments, while the vertical axis is the difference between the two. The solid horizontal line is the average difference and the dashed horizontal lines show estimates of the limits of agreement. Error bars on those lines indicate uncertainty surrounding those estimates. The average difference between the two devices was $1.5\times 10^{-4}\;{\mathrm{mm}}^{-1}$ in $\mu_{a}$ and $0.017\;{\mathrm{mm}}^{-1}$ in $\mu_{s}^{\prime }$, respectively. The limits of agreement in $\mu_{a}$ and $\mu_{s}^{\prime }$ were −0.0038 and 0.0041±0.0007 and −0.06 and 0.09±0.02, respectively.

Bland–Altman analysis^43^ was performed to compare the FLIR openSFDI system to the commercial device. We found that the average difference between the two devices was $0.00015\;{\mathrm{mm}}^{-1}$ in $\mu_{a}$ and $0.017\;{\mathrm{mm}}^{-1}$ in $\mu_{s}^{\prime }$. Importantly, the accuracy was maintained across the entire range of $\mu_{a}$ and $\mu_{s}^{\prime }$ values tested. The limits of agreement in $\mu_{a}$ were −0.0038±0.0007 and 0.0041±0.0007 and the limits of agreement in $\mu_{s}^{\prime }$ were −0.06±0.02 and 0.09±0.02, respectively. In Bland–Altman analysis, the limits of agreement indicate the region where difference between the two instruments will fall 95% of the time. That is, if 100 measurements were made on the same samples with openSFDI and the commercial instrument, the differences between about 95 of those measurements will fall within the limits of agreement. However, there is uncertainty associated with the limits of agreement themselves as indicated by the error bars in Fig. 5. The importance of this uncertainty is illustrated in Fig. 5 where there are 81 points per plot. In the absorption panel, eight points (9.8%) fall outside the estimated limits of agreement, while only three points (3.7%) fall outside the region bounded by the uncertainty of the limit of agreement estimates. Looking at the limits of agreement in isolation can give an inaccurate picture of the differences between the two instruments.^44^

### 5.2 Precision

**Fig. 6:**
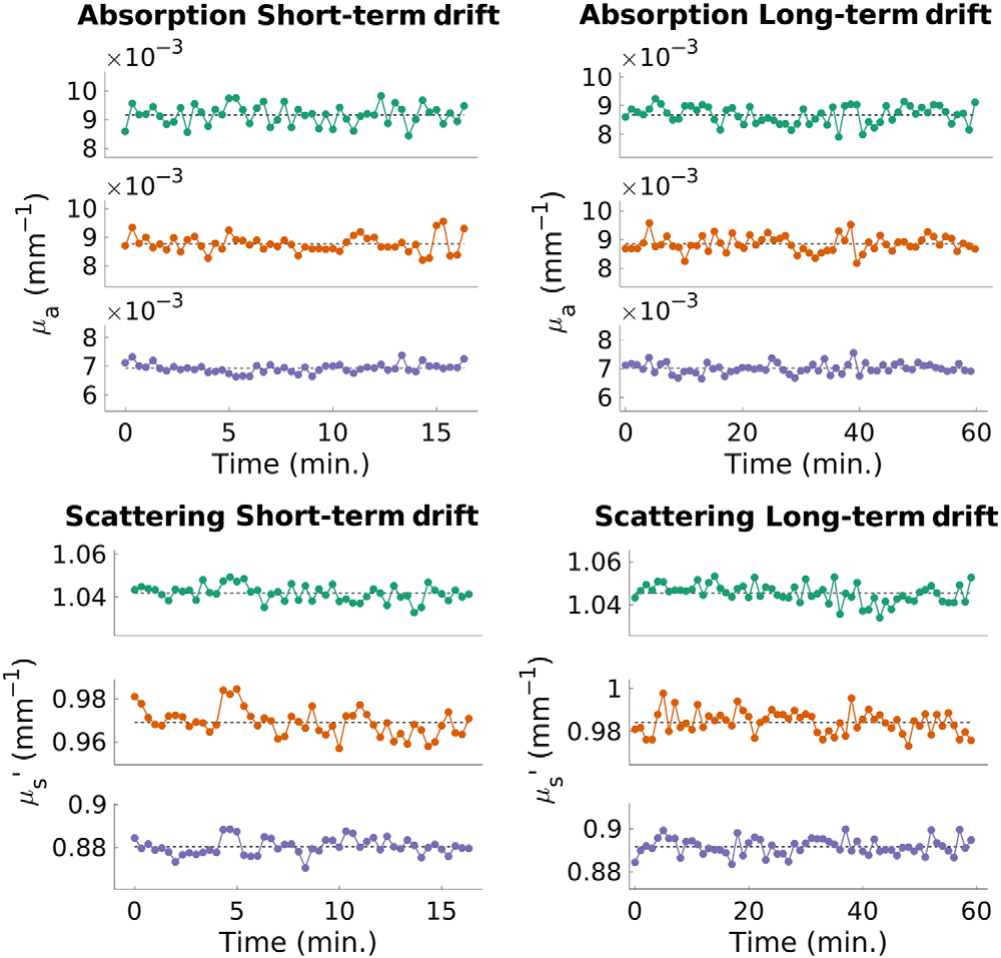
Results from a 15-min drift measurement (left column) and a 60-min drift measurement (right column) showing the stability of the $\mu_{a}$ (top row) and $\mu_{s}^{\prime }$ (bottom row) measurements. The subplots in each panel correspond to the different wavelengths with the top plot representing 660 nm, the middle plot representing 735 nm, and the bottom plot representing 865 nm. Dashed lines represent the means of each measurement. The average standard deviation of $\mu_{a}$ was $0.0004\;{\mathrm{mm}}^{-1}$ and the average standard deviation of $\mu_{s}^{\prime }$ was 0.005.

To evaluate the precision of openSFDI, we conducted repeated measurements of the same optical phantom. Two different scenarios were explored. In the first scenario, SFDI measurements were repeated every 20 s over a 15-min period. This corresponds to a situation in which time resolution is important (e.g., measurements during a vascular occlusion). In the second scenario, SFDI measurements were repeated every minute for 1 h. This corresponds to a situation in which slower changes in optical properties are expected. Figure 6 demonstrates that there is no trend in either $\mu_{a}$ or $\mu_{s}^{\prime }$ that would indicate drift over time. Rather, the differences in $\mu_{a}$ and $\mu_{s}^{\prime }$ move randomly about the mean. Overall, the average standard deviation of the $\mu_{a}$ measurements across all wavelengths was $0.0002\;{\mathrm{mm}}^{-1}$ in both the shorter drift measurement and the 1-h measurement. In $\mu_{s}^{\prime }$, the average standard deviation was $0.005\;{\mathrm{mm}}^{-1}$ during the short duration measurement and $0.004\;{\mathrm{mm}}^{-1}$ over the 1-h measurement.

### 5.3 Extensions

The openSFDI system was designed to be easily modified and extended. Wherever possible, modular components were chosen to allow for modifications to the system to be made. In this section, we discuss several potential openSFDI modifications.

#### 5.3.1 Wavelengths

The three wavelengths described in this work (660, 735, and 865 nm) were chosen for their availability and sensitivity to oxyhemoglobin and deoxyhemoglobin.^45^ However, different wavelengths can be used to target different chromophores. Altering the wavelengths of the openSFDI system can be accomplished by swapping one of the LED assemblies and (if necessary) changing the dichroic mirrors.

#### 5.3.2 Profilometry

**Fig. 7:**
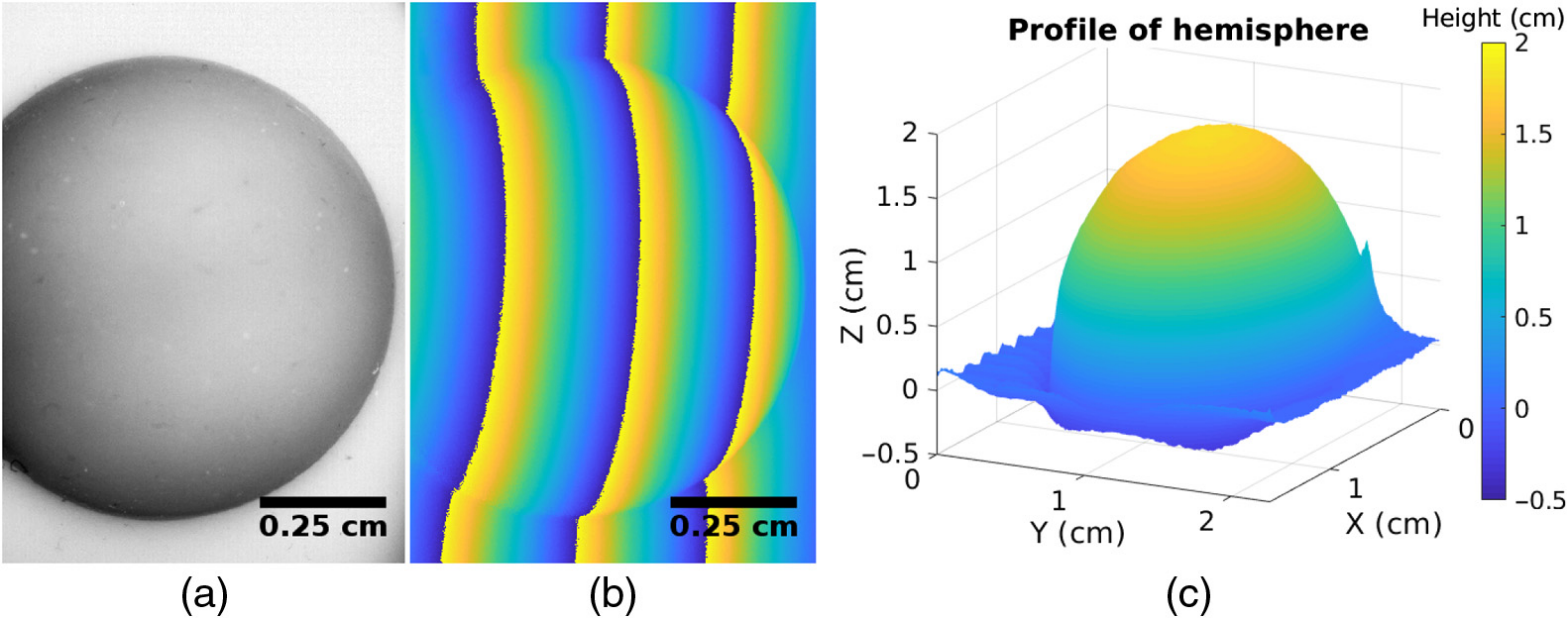
(a) Raw intensity image of a 1.75-cm-diameter hemispheric phantom showing how the intensity changes as a function of height. (b) Wrapped phase image of the same phantom. The effect of height can be clearly seen by the curve of the phase isolines. (c) Three-dimensional rendering of the object’s profile following phase unwrapping and calibration of panel (b).

The profile of the sample can be used to account for portions of the sample that are closer or farther away from the camera, providing more accurate optical properties for samples with irregularly geometry.^46^^,^^47^ Briefly, this is accomplished by collecting measurements of a phantom with known optical properties at different heights. From these measurements, a linear fit comparing pixel intensity to height can be derived for each pixel. Once the object’s profile is known, this linear fit can be used to add or subtract intensity based on the calibration measurement. Profilometry can be performed using a phase-stepping technique in which sinusoidal patterns are projected onto the sample. The unwrapped phase image maps directly to the object’s profile.^46^ Figure 7 shows an example of profilimitry conducted with the openSFDI system on a hemispheric phantom.

#### 5.3.3 Optics selection

The openSFDI system also provides flexibility in the optics that govern projection and detection of patterns. The suggested design results in a field of view that is ∼4×8  cm. The field of view can be adjusted by changing the height of the system, increasing the diameter of the relay mirror, or changing the projection lens.

The basic design calls for a standard CMOS camera that can be interfaced with a variety of lenses to adjust the field of view. In our lab, we have tested a variety of other cameras and imaging lenses. We have validated the performance with both CMOS and sCMOS cameras (Fig. 4) through a variety of lenses. We have also successfully modified the openSFDI system to use a microscope objective as the imaging lens. Changing the field of view will also impact the intensity of the light hitting the sample. To ensure good signal-to-noise ratio of the images, the LED power, exposure times, or aperture of the imaging lens may be necessary.

## 6 Discussion

The openSFDI system described here and on the companion website provides a low-cost and step-by-step way for researchers to explore SFDI as a potential tool for new applications.^28^ However, as with any imaging modality, there are particular challenges that can limit the accuracy of SFDI imaging. While touched on in the body of the paper, there are a few points that deserve more careful attention.

DMDs are inherently binary, with each mirror being switched fully on or fully off. Shades of gray are realized by pulse-width modulation (PWM) of the mirrors. The period of the PWM is known as the refresh rate of the DMD which is 16.7 ms for the LC4500. When imaging, care should be taken not to reduce the exposure time below the refresh rate as it will result in a distorted image. Also, we have observed in some SFDI systems that using an exposure time that is an integral multiple of the refresh rate can reduce the presence of artifacts in the demodulated images. However, no effort to match these values was made in this work.

The openSFDI does not include source code for processing. This is by design so that users can gain experience in developing each step in the processing chain for their particular application. We note that special care is warranted in the matter of calibration. The accuracy and precision of the calibration phantom optical properties will affect subsequent downstream processing steps and overall SFDI results. Though it is beyond the scope of this paper, we encourage users to consider how potential errors in the calibration phantom optical properties propagate to the final images.

## 7 Conclusion

In this paper, we have outlined the design and performance of openSFDI, an open source hardware SFDI system. The companion website to this paper^28^ provides a complete materials list as well as step-by-step instructions for constructing and aligning the openSFDI platform. The website also provides software written in LabVIEW that enables rapid acquisition of SFDI datasets and detailed instructions for developing a data-processing pipeline. Here we described three different openSFDI systems built at two different locations, each of which had excellent accuracy and precision in optical property extractions and was able to measure oxyhemoglobin and deoxyhemoglobin concentrations in biological samples. We hope that the openSFDI platform will lower the barrier-to-entry for SFDI and help make it more accessible to researchers around the world.
